# Assessment of the Gail Model in Estimating the Risk of Breast Cancer: Effect of Cancer Worry and Risk in Healthy Women

**DOI:** 10.31557/APJCP.2019.20.6.1765

**Published:** 2019

**Authors:** Abdulbari Bener, Cem Cahit Barışık, Ahmet Acar, Yaşar Özdenkaya

**Affiliations:** 1 *Department of Biostatistics and Medical Informatics, Cerrahpaşa Faculty of Medicine, Istanbul University, *; 3 *Istanbul Medipol University, International School of Medicine, *; 4 *Department of Radiology and Pathology, *; 5 *Department of Surgery, Medipol School of Medicine, Istanbul Medipol University, Istanbul, Turkey, *; 2 *Department of Evidence for Population Health Unit, School of Epidemiology and Health Sciences, University of Manchester, Manchester, UK. *

**Keywords:** Breast cancer, Gail model risk assessment, lifestyle, predictor, risk factors, consanguinity, Turkish women

## Abstract

**Background:**

There has been substantial interest in developing methods to predict the risk of breast cancer. The Gail model is one the first model have been widely used to identify women at higher risk of breast cancer.

**Aim:**

This study aimed to determine the 5-year and the general life-time risk of breast cancer and also to determine breast cancer predictors in women using the Gail model.

**Methods:**

We used the Gail model to estimate the risk of breast cancer in female Turkish outpatients aged above 35 years in this cross-sectional study. Age, life-style habits, breast-feeding duration, family history of breast cancer, and body mass index were compared between high and low-risk subjects. We have performed the Patient Health Questionnaire 9-item (PHQ-9) and the Generalized Anxiety Disorder 7-item (GAD-7) tools on patients regarding depression and anxiety. We also assessed the association of these covariates with the estimated risk of breast cancer in multivariate linear regression analysis.

**Results:**

We enrolled 1065 subjects with a mean age of 52.9 ± 8.4 years. The mean of the five-year risk for breast cancer was 1.33%±0.6. Meanwhile, the mean of lifetime risks for breast cancer was 10.15%±3.18, respectively. Nearly one-third of the participants had one child, 55.9% had breast-fed their children more than six months. Meanwhile, 18.5% of the subjects had a high depression score, 15.2% had a high anxiety score. Higher age, age at first birth, and parity; lower age at menarche; presence of menopause and family history of breast cancer were higher in the high-risk group. Higher age, and age at first birth; lower age at menarche; family history of breast cancer, presence of menopause, and parity were independently associated with higher breast cancer risk.

**Conclusion:**

We identified certain risk factors for breast cancer in our study population and Gail model is a reliable and useful breast cancer risk prediction model for clinical decision-making. This study contributes to the body of evidence in order to facilitate early detection and better plan for possible malignancies in Turkish population.

## Introduction

Breast cancer is the most commonly diagnosed cancers in women worldwide and its account for 29% of all new cancers in women at 2015 (Siegel et al., 2015) with high morbidity and mortality rates. Risk assessment tools estimating the individual’s absolute risk for developing breast cancer and identifying the women at high level of risk are crucial for decision-making about prevention and screening (Globocan 2012; World Health Organization, WHO, 2014). 

Among Turkish women, breast cancer is the most common cancer and the most common cause of cancer related death (Ministry of Public Health, Turkey, Ulusoy et al., 2010; Erbil et al., 2015). Investigators have suggested various models to estimate the risk of breast cancer using several risk factors identified in epidemiological studies (Gail et al., 1989). Among these tools, the Gail model, which utilizes the risk factors identified in the Breast Cancer Detection Demonstration Project (BCDD) in 1989, is the most commonly used one (Gail et al., 1989; Ulusoy et al., 2010; Erbil et al., 2015) and has been modified (Costantino at al., 1999). About 5% to 10% of breast cancers are thought to be hereditary, caused by abnormal genes passed from parent to child (Claus et al., 1994; Bener et al., 2010). Genetic factors play an important role in the pathogenesis of breast cancer and family history of breast cancer is a well-known risk factor (Bener et al., 2010, Bener et al., 2017). Given the benefits of early diagnosis and prevention of breast cancer, implementation of breast cancer screening is crucial, especially for “high-risk” women (Tyrer et al., 2005; Gail et al., 2007; Adams –Campbell et al., 2009; Pace and Keating 2014). The Gail model incorporates six breast cancer risk factors, namely: age, age at menarche, age at first live birth, number of breast biopsies, history of atypical hyperplasia, and number of first-degree relatives with breast cancer (Gail et al., 1989; Claus et al., 1994; Costantino at al., 1999).

One of the advantages of Gail model (Park et al., 2013; Khazaee-Pool et al., 2016; Mirghafourvand et al., 2016) is the extensive validation in various female populations for more than two decades. We aimed to estimate 5-year and life-time risk of breast cancer and determine risk factors associated with higher breast cancer risk in Turkish population.

## Introduction

This cross sectional study was conducted among the outpatients patients registered in internal medicine clinics, surgery, ophthalmology of outpatient clinics, Obstetrics - Gynecology - Medipol International Health Centers during the study period from June 2016 to March 2018 among Turkish citizens above 35 years of age. IRB ethical approval for this study was provided by the Istanbul Medipol University and conducted in accordance with the Declaration of Helsinki. All participants gave consent and approved prior to inclusion in the study. 

The sample size calculation was based on previous studies that determined the prevalence of Breast cancer in Turkey (Ulusoy et al., 2010; Erbil et al., 2015) to be between 25%-27%, with the 99% confidence interval and with 2.5% error of estimation. The minimum sample size for the current study was 1,500. Subjects were recruited by the systematic 1-in-2 sampling procedure. During the study period, a representative sample of 1,500 women aged 35 years and older was selected, 1,065 (71.0%) subjects gave consent. Meanwhile, 435 women either refused or were not available to take part in the study due to personal reasons and lack of time.

We have calculated the five-year and life-time risk for breast cancer using Breast Cancer Risk Assessment Tool (BCRAT, 2018) which is based on the Gail model (Gail et al., 1989; National Cancer Institute 2018). According to the Gail model, women with the breast cancer risk of >1.66% were considered as high-risk according to the estimated 5-year breast cancer- risk assessment. The Gail Risk Assessments Tool is useful to guess the approximate number of women with a lifetime risk of ≥15% in the general population (Gail et al., 1989; Pace and Keating, 2014).

We calculated the five-year and life-time risk for breast cancer using Breast Cancer Risk Assessment Tool (BCRAT) which is based on the Gail model **(**Mirghafourvand et al., 2016). BCRAT is an interactive tool which estimates 5-year and lifetime risk for breast cancer. Stratification of risk level is one of the main targets of risk assessment tools which facilitates screening decision and clinical management. According to the Gail model, while a five-year breast cancer risk of less than 1.66% indicates low risk, an estimated risk of 1.66% or higher suggests high risk (National Cancer Institute, 2018). We determined the mean 5-year and lifetime risks of our study population and classified the subjects as low- or high-risk if they had a lower or higher risk than the mean of the study population respectively. In this context, a five-year breast cancer risk of 1.33% or higher and a life-time breast cancer risk of 10.15% or higher indicated high-risk for this study.

*Patient health questionnaire 9-item (PHQ-9)*

Understanding psychological factors relevant to women’s participation in breast cancer screening is essential to ensuring that clinical and public health policies and interventions improve breast cancer morbidity and mortality. The PHQ-9 contains nine items referring to criteria for depression (Kroenke et al., 2001) and the answers refer to the past two weeks. The PHQ-9 has been shown to have good reliability and validity (Kroenke et al., 2001). Overall, the PHQ-9 items showed good internal (Cronbach’s alpha 0.85) reliability. A cut-off score of 10 was established for the PHQ-9 (sensitivity 87%, specificity 89%), correctly classifying 86% of patients with current depression. 

*Generalized anxiety disorder 7-item (GAD-7)*

The GAD-7 (Spitzer et al., 2006) tool conducted on subjects are asked how often, bothered by each of the seven core symptoms of generalized anxiety disorder and cut-off point of 10 to describe clinical symptoms of anxiety in the sample. The GAD-7 has been shown to have good reliability and validity (Spitzer et al., 2006). Cronbach’s alpha in the current study was 0.87.

Statistical analysis and comparisons are based on Independent Student-t test, Chi-square and Fisher’s exact test. Multiple linear stepwise regression analysis performed to predict the 5-year and lifetime breast cancer risk. The level p<0.05 was considered as the cut-off value for significance. 

## Results

We assessed content validity, face validity and reliability of the questionnaire in 100 subjects. These tests demonstrated a high level of validity and high degree of reproducibility (kappa = 0.87). We tested the validation of the pilot survey instruments in 100 subjects. We demonstrated adequate scale reliability with Cronbach’s alpha coefficient values higher than 0.70. The Cronbach’s alpha for overall internal reliability was 0.87.

Table 1 shows the socio-demographic features of the study population. The mean age was 52.94 ± 8.48 years. Among the study population, 87.9% were married, 56.1% were housewives, 10.2% were illiterate, and 23.9% were university graduates. Majority of participants (57.6%) had experienced menarche between the ages 12 and 13 years. Nearly two thirds of the study population (66.4%) were post-menopausal., Rates of cigarette smoking (17.6%) and sheesha smoking (10.0%) were low. 

Table 2 demonstrates life-style and clinical features of the study population. Among the study population, 26.5% walked 30 minutes per day and 11% walked 60 minutes per day. Frequency of overweight was 45.6% and 28.9% were obese. Nearly one-third of the participants had one child. Most of the participants (55.9%) had breast-fed their children more than six months. While 18.5% of the subjects had a high depression screen score, 15.2% had a high anxiety screen score.

The mean of the five-year risk for breast cancer was 1.33%±0.6. Meanwhile, the mean of lifetime risks for breast cancer was 10.15%±3.18, respectively. Table 3 demonstrates socio-demographic features of high-risk and low-risk women in comparison according to their five-year and life-time breast cancer risk. There were significant differences between high-risk and low-risk groups according to five-years and life-time breast cancer risk estimation such as age, age at menarche, age at first birth, family history of cancer, menopausal status, parity (only for five-year risk), BMI, occupation, and level of education according to five-year risk estimation; and age, age at menarche, age at first birth, family history of cancer, menopausal status, and level of education according to life-time risk estimation. 

While age, age of first birth, family history, menopausal status, and parity were positively related with five-year breast cancer risk, age at menarche was inversely related with five-year breast cancer risk according to multivariate linear regression analysis (Table 4). Variables positively associated with life-year breast cancer risk were age of first birth, family history, menopausal status, and parity; while variables inversely related with life-year breast cancer risk consisted of age and age at menarche according to multivariate linear regression analysis.

**Table 1:** Socio-Demographic Characteristics of Breast Cancer Patients (N= 1,065)

Age [Mean ± SD]	n	%
	52.94 ± 8.48	Range 35-65
Age groups		
35-45	226	21.2
46-55	377	35.4
56-65	462	43.4
Age at menarche		
9-11 Years old	186	17.5
12-13 Years old	626	58.8
>14 Years old	253	23.8
Menopausal		
Pre-menopausal (non-menopause)	358	33.6
Post-menopausal (menopause)	707	66.4
Marital status		
Single	62	5.8
Married	936	87.9
Widow/divorce	667	6.3
Education level		
Illiterate	109	10.2
Primary	190	17.8
Intermediate	217	20.4
Secondary	303	28.5
University or higher	446	23.1
Occupation		
Housewife	597	56.1
Sedentary/Professional	175	16.4
Clerk/Officer/Administrator	163	15.3
Businesswomen	67	6.3
Police / Army/Security Force	63	5.9
Household Income		
Low	294	27.6
Medium	435	40.8
High	336	31.5
Cigarette smoking		
Never	877	82.3
Current smoke	141	13.2
Ex-smoker	47	4.2
Sheesha Smoking		
Yes	106	10.0
No	959	90.0

**Table 2:** Life-Style and Clinical Characteristics of Study Sample (N=1,065)

Variables	n (%)
Physical activity walking per-day	
30 Minutes	282 (26.5)
60 Minutes	118 (11.0)
None	665 (62.5)
Body Mass index Group-BMI (Kg/m^2^)	
20-24.99 Kg/m^2^ (Normal)	272 (25.5)
25-30 Kg/m^2^ (Overweight)	485 (45.6)
>30 Kg/m^2^ (Obese)	308 (28.9)
Infertility	
Yes	72 (6.8)
No	993 (93.2)
Parity	
None	82 (7.7)
<3 children	575 (54.0)
>3 children	408 (38.3)
Breast-feeding	
Yes	877 (82.3)
No	188 (17.7)
Breast-feeding duration	
6 months	354 (33.2)
> 6 months	711 (66.8)
Consanguineous parents	
Yes	59 (5.5)
No	1006 (94.5)
First degree family cancer history	
Yes	62 (6.8)
No	1003 (94.2)
Family cancer history more than one	
Yes	71 (6.7)
No	994 (93.3)
Mammography screening	
Yes	89 (8.4)
No	976(91.6)
Depression PHQ-9 score *	
Score>10	197 (18.5)
Score ≤10	868 (81.5)
Anxiety GAD-7 score **	
Score >10	162 (15.2)
Score ≤ 10	903 (84.8)

* Patients were considered depression “screen-positive” if their PHQ-9 score was >10; ** Patienats were considered anxiety “screen-positive” if their GAD-7 score was > 10.

**Table 3:** Socio-demographic of Patients with Breast Cancer Risk Using Gail Model (N= 1,065)

	5-Year Risk Mean (1.33±0.60 )			Lifetime Risk Mean (10.15±3.18)		
	Low Risk	High Risk n (%)	p value n (%)	Low Risk n (%)	High Risk n (%)	p value n (%)
Age Groups						
35-45	216 (38.4)	10 (2.0)		72 (12.3)	154 (32.0)	
46-55	239 (42.5)	138 (27.5)	<0.001	186 (31.9)	191 (39.6)	<0.001
56-65	108 (19.1)	354 (50.5)		325 (55.8)	137 (28.4)	
Age at Menarche						
9-11	76 (13.5)	110 (28.3)		86 (14.8)	100 (20.7)	
12-13	339 (63.2)	287 (57.2)	<0.001	375 (57.5)	291 (60.4)	<0.001
14	148 (26.3)	105 (20.9)		162 (27.8)	91 (18.9)	
Age at First Birth						
<20	101 (12.9)	10 (3.2)		106 (18.2)	11 (2.3)	
20-24	171 (30.4)	56 (11.2)	<0.001	185 (33.4)	32 (6.6)	<0.001
25-29	190 (33.7)	149 (29.7)		204 (35.0)	135 (28.0)	
30	101 (12.9)	281 (56.0)		78 (13.4)	304 (63.1)	
Family History						
Yes	42 (7.5)	63 (12.5)	<0.001	50 (8.6)	61 (12.7)	<0.001
No	521 (92.5)	439 (87.5)		533 (91.4)	421 (87.7)	
Menopausal						
Pre-menopausal	315 (56.0)	43 (8.6)	0.419	134 (23.0)	224 (46.5)	<0.001
Post-menopausal	248 (44.0)	459 (91.4)		449 (77.0)	258 (53.5)	
Breast-feeding						
<6 months	193 (34.3)	161 (32.1)	0.474	196 (33.6)	158 (32.8)	0.412
6 months	370 (65.7)	341 (67.9)		387 (66.4)	324 (67.2)	
Consanguinity						
Yes	35 (6.2)	24 (4.8)	0.043	39 (6.7)	20 (4.1)	0.195
No	518 (93.8)	478 (95.2)		544 (93.3)	462 (94.9)	
Parity						
3 Children	368 (65.4)	289 (57.6)	<0.001	357 (61.2)	300(76.1)	0.267
>3 Children	195 (34.6)	213 (42.4)		226 (38.8)	182 (23.9)	
BMI						
20-24.99 Kg\m^2^	158 (28.1)	114 (22.7)		136 (23.3)	136 (28.2)	
25-30 Kg\m^2^	232 (41.2)	253 (50.4)	0.010	283 (48.5)	202 (41.9)	0.071
>30 Kg\m^2^	173 (30.7)	135 (26.9)		164 (28.1)	144 (29.9)	
Breast Biopsies						
Yes	6 (1.1)	7 (1.4)	0.416	7 (1.2)	6 (1.2)	1.000
No	557 (98.9)	495 (98.6)		576 (98.9)	476 (98.8)	
Sheesha Smoking						
Yes	53 (9.4)	53 (10.6)	0.301	52 (8.9)	54 (11.2)	0.219
No	510 (90.6)	449 (89.4)		531 (91.1)	428 (88.8)	
Cigarette smoking						
Yes	93 (16.5)	95 (18.9)		100 (17.2)	88 (18.3)	0.845
No	470 (83.5)	407 (81.1)		483 (82.8)	394 (81.7)	
Occupation						
Housewife	295 (52.4)	302 (60.2)		324 (55.6)	273 (56.6)	
Sedentary\Professional	119 (21.1)	56 (11.2)		84 (14.4)	91 (18.9)	0.114
Clerk\Administrator	113 (20.1)	113 (22.5)	<0.001	138 (23.6)	88 (18.3)	
Businesswomen	36 (6.4)	31 (6.2)		37 (6.3)	30 (6.2)	
Education Level						
Illiterate	44 (7.8)	65 (12.9)		73 (12.5)	36 (7.5)	
Primary	96 (17.1)	94 (18.7)		95 (16.3)	95 (19.7)	
Intermediate	114 (20.2)	103 (20.5)	0.030	131 (22.5)	86 (17.8)	0.00vf9
Secondary	156 (27.7)	147 (25.3)		160 (27.4)	143 (29.7)	
University or higher	153 (27.2)	83 (18.5)		124 (21.3)	122 (25.3)	

**Table 4:** Regression Results for 5-year and Lifetime Gail Risk

Independent Variables	Coefficient	Standard Error	t	p-value
5-Year Risks				
Constant	-0.176	0.36	-1.298	0.195
Age	0.032	0.001	25.310	<0.001
Age at Menarche	-0.034	0.007	-4.511	<0.001
Age of First Birth	0.204	0.011	18.897	<0.001
Family History	-0.134	0.035	-3.732	<0.001
Menopausal	0.529	0.028	3.499	<0.001
Parity	0.076	0.020	2.878	0.004
Lifetime Risks				
Constant	2.476	0.151	16.383	<0.001
Age	-0.025	0.002	-10.154	<0.001
Age at Menarche	-0.034	0.008	-4.423	<0.001
Age of First Birth	0.273	0.011	23.761	<0.001
Menopausal	0.261	0.031	-8.328	<0.001
Family History	-0.108	0.038	-2.861	0.004
Parity	0.056	0.024	2.319	0.021

## Discussion

The Gail model (Gail et al 1989; Costantino et al., 1999; Gail et al., 2007; Adams –Campbell et al., 2009).is most commonly used in estimation of risk of breast cancer. It is important to accurately assess breast cancer risk to take preventive measures and make decision on screening procedures. Breast cancer is the most common type of cancer and accounts for the greatest number of cancer-related death among women in Turkey (Ulusoy et al., 2010; Erbil et al., 2015). The Gail model might overestimate breast cancer risk for Asian and Middle-Eastern women. Thus, it is needed to revalidate the Gail model in these populations. In this study, we aimed to revalidate the Gail model using data of Turkish women, and subsequently update and revalidate the Turkish version of the Gail model using our data. The results of our study indicate that Turkish version of the Gail model is valid and provides clinically relevant information. Age, age of first birth, age at menarche, family history, menopausal status, and parity seems to be independently associated with estimated breast cancer risk.

In Iran study (Khazaee-Pool et al., 2016), the mean five-year risk of breast cancer for all participants was 1.608±0.729% (range 0.2±13.8%).The mean lifetime risk of breast cancer was 11.705±3.91% (range 0.6 54.7%).Similarly, in Turkey, analysis revealed that the mean of the five-year risk for breast cancer was 1.33%±0.6. and meanwhile, the mean of lifetime risks for breast cancer was 10.15%±3.18, which is consistent with the Iranian results.

The best-known statistical model available for predicting an individual woman’s chance of developing breast cancer is that derived using information from regularly screened Caucasian women from the United States participating in the Breast Cancer screening and detection project (Gail et al 1989; Costantino et al., 1999; Gail et al., 2007; Adams –Campbell et al., 2009). The Gail model might overestimate the risk of and development of breast cancer in other populations in which other races predominate (Novotny et al., 2006; Andreeva and Pokhrel 2013; Wang et al., 2018). However, utilization of the Gail model for Turkish population and other populations with white women predominance might provide more accurate estimations (Tice et al., 2005; Min et al., 2014; Wang et al., 2018). Accordingly, the five-year and life-time estimated risk for breast cancer was not higher in our study than different studies (Tice et al., 2005; Min et al., 2014; Khazaee-Pool et al., 2016, Wang et al., 2018). Unfortunately, the Gail model for breast cancer risk prediction of consanguineous marriages for fist degree and second-degree relatives is not included as a risk factor. On the other hand, the Claus model (Claus et al., 1994) takes presence of both first and second-degree relatives with breast cancer and their age at diagnosis into account as risk factors. BRCAPRO model utilizes Bayesian statistics and Mendelian approaches and considers family history of bilateral breast cancer and ovarian cancer as risk factors (Bener et al., 2010; 28-30). The Tyrer-Cuzick (Tyrer et al., 2004) estimated 10-year risk model which also provides information about the possibility of a breast cancer susceptibility gene mutation (Berry et al., 2002; McPherson et al., 2000). 

Estimation of breast cancer risk is essential in the management of screening and prevention of this cancer. Closer screening may be beneficial in high-risk individuals. Screening with mammography has provided an important decrease in breast cancer mortality in women over 50 years old in Western countries (Gail et al., 1989; Claus et al., 1994; Costantino at al., 1999; McPherson et al., 2000; Berry et al., 2002). However, the advantage of screening is less clear for younger women. It would be better to individualize breast cancer screening for women under 50 years old. In this context, risk estimation tools such as the Gail model may prove beneficial., Although, applying risk assessment models can help healthcare providers to calculate a women’s risk of developing breast cancer. It has been recommended that women should have annual mammograms starting at age 40 (Min et al., 2014). In fact, women with a higher risk of developing breast cancer should get extra screening procedures; as well as they might also get beginning screening at an earlier age with more repeated periods (McPherson et al., 2000; Park et al., 2013; Bener et al., 2017).

To our knowledge, the Gail model has not been validated in a population with as large sample size as our study in Turkish women. Nevertheless, we examined the effect of factors that did not take place in the Gail model (i.e., BMI, consanguinity, duration of breast feeding, and menopausal status). In line with the findings of several studies (Gail et al., 1989; Claus et al., 1994; Costantino at al., 1999; McPherson et al., 2000; Gail 2007; Adams-Campbell et al., 2009), although our study suggested that the breast cancer risk was high in older age, presence of menopause, lower age at menarche, higher age at first birth, women with family history of breast cancer.

Furthermore, PHQ -9 and GAD-7 appears to be a reliable and valid instrument that can be used to diagnose major depression (18.5%) and anxiety (15.2%) among Turkish women. This is consistent the with prevalence rate of major depression and anxiety observed rates 15.4% and 14.4% in United States among women (Elrashidi et al., 2018), also similarly study reported for the depression 14.7%, and anxiety 10.1% conducted in Latvia women (Ivanovs et al., 2018).

In conclusion, breast cancer is major public health issue in Turkey. For preventing and screening of breast cancer, estimation of risk of breast cancer in Turkish population is crucial., Our study indicates that Gail model is a reliable and useful breast cancer risk prediction model for clinical decision-making. The Gail et al., model fits very well in this sample in terms of predicting numbers of breast cancer cases in specific risk factor strata but had modest discriminatory accuracy at the individual level. Our study may prove beneficial in the management of breast cancer counseling provided that the results of our study validated in prospective studies.

## Contributors

AB, CCB and YÖ are designed and supervised the study and were involved in data collection, statistical analysis the writing of the paper. AA was involved in interpretation of data and writing manuscript. All authors approved the final version of this manuscript.

## Ethics Committee Approval

Ethics committee approval was received for this study.

## Informed Consent

Informed consent was obtained for this study.

## Financial Disclosure

The authors declared that this study has received no financial support.

## Competing interests

We have no financial interest to declare.
